# On the Influence of the Systems Recently Adopted in Various Prisons upon the Health of Their Inmates

**Published:** 1841-10

**Authors:** 


### Art. XV.

*Om de Sanitaire Forholde i Fængsler efter nyere Systemer.* Ved Professor Fred. Holst, m.d.—*Christiania*, 1840. 8vo, pp. 30.

*On the Influence of the Systems recently adopted in various Prisons upon the Health of their Inmates.* By Professor Fred. Holst, m.d. —*Christiania*, 1840.

At the meeting of the Scandinavian Naturalists in Copenhagen in July, 1840, Professor Holst of Christiania gave a long and interesting account of the sanitary results which have been obtained under those systems of prison discipline which have recently been adopted in Europe and America. The learned Professor reminds his assembled colleagues that he addressed them upon this subject at the meeting of the previous year in Gothenborg, and hopes that he then fully proved that the Philadelphia system, as adopted at Cherry Hill (United States) and elsewhere, was that which presented the most advantageous results, both in a moral and economical point of view. The object of again bringing this subject before the meeting was to discuss the important question of the effects of this and of other systems upon the health of the inmates of those prisons where they have been adopted. Both the Auburn and

Philadelphia systems have been decried as more injurious to the bodily and mental powers of the prisoners than any of the older plans of discipline in prisons, and this blame has in particular been attached to that of Philadelphia. Two years ago these heavy charges obtained additional weight from being corroborated by two individuals whose position gave no small increase to the value of their testimony, viz. the physicians Gosse and Coindet of Geneva.

The efficacy of the sanitary regulations of a prison will be best determined by the relative degrees of sickness and mortality within its walls. But in this regard we experience the greatest difficulty in obtaining correct reports. Many individuals are entered upon the sick-list by one physician who by another would be passed by as healthy; and again we must take into consideration the average duration of each individual's term of punishment, for unquestionably the longer an individual is confined within the walls of a gaol the greater is the probability that his health will become seriously affected.

Having adverted to these sources of error, Professor Holst presents us with a table of the relative mortality in those prisons of Europe and America which are regulated on the plans of Auburn and Philadelphia, as well as in those conducted on the older systems. From this table it appears that the mortality, *in proportion to the general mortality of the country*, has been always greatest in the prisons conducted on the older plans, and least in those which are regulated according to the system of Philadelphia. But on referring to the table we find the highest *real* mortality to occur in Cherry Hill prison (Philadelphian system), the deaths being there as 1 in 26, while in the Glasgow Bridewell, under the same system of discipline, the mortality was only as 1 to 55. Professor Holst maintains, however, that we cannot form any accurate conclusions from the high rate of mortality in the prisons of Cherry Hill and Pittsburg, as in these two places of confinement is to be found a much greater proportion of the coloured population of the country than in the other prisons of the United States.

Out of the results of these investigations Professor Holst has constructed another table, showing the excess of mortality in prisons under the three different systems referred to, over the general mortality of the country.

| Mortality in Prisons. | Mortality of the general population of the country. | Excess of Mortality in Prisons. |
|---|---|---|
| Previous Systems.<br>1 : 27 = 3·70 per cent. | 1 : 42 = 2·38 per cent. | 1·32 per cent. |
| Auburn System.<br>1 : 35 = 2·86 | 1 : 47 = 2·13 | 0·73 |
| Philadelphia System.<br>1 : 40 = 2·50 | 1 : 47 = 2·13 | 0·37 |

It is obvious from this last table, which admits of a far more practical application than the former one, that the causes of this unequal ratio of mortality must be sought for in the difference of the systems themselves.

It cannot be expected that individuals deprived of their liberty by being confined within the walls of a prison should enjoy such perfect health as the free population of the country.

**"Let us then suppose," says Prof. Holst, "that all the necessary conditions for preserving the health of the prisoners have been fulfilled, and this under the two recent systems is unquestionably the case, as well as in some other gaols, where the new discpline has not as yet been adopted. . . . . . . . . . . But in these last the prisoner enjoys many advantages and privileges which are either absolutely prohibited, or conceded on a much more limited scale, under the recent systems of Auburn and of Philadelphia. . . . . . . . . . . For instance the prisoner in the unreformed gaols passes both the day and the night in company with his fellow-captives; he is permitted in many cases to converse with his friends and with his relatives, and to take abundant exercise in the open air, all which advantages are highly valued by those in confinement." (p. 8.)**

Professor Holst then proceeds to lay before his auditors a short account of the systems of Auburn and Philadelphia. Under the Auburn system, each prisoner is shut up at night in a solitary cell, and during the day the inmates of the prison labour indeed in community, but the strictest silence is enforced. Besides this they are daily permitted to pass a certain time in the open air. Silence is in fact the groundwork of this system, and the observance of this law is considered of the greatest importance. But offences against this rule are naturally numerous, and the minds of the prisoners are exasperated by repeated and severe punishments for such transgressions. In the House of Correction at Geneva, where a modification of the Auburn system is pursued, various privileges were for some years conceded to the inmates, by which indeed the number of punishments for offences within the prison walls was considerably diminished, but relapses into crime on being released became in a corresponding degree more frequent. Since 1833 a severer system has been adopted in this prison, and the average of relapses has diminished from 33 to 7½ per cent.

The main principle of the Philadelphia system consists in the total separation of the prisoners one from another. Both day and night they remain perfectly apart; but silence is not enjoined, and their cells are considerably larger than those under the Auburn system. In the House of Correction at Cherry Hill (United States), the prisoner is *never* permitted to leave his cell; in Glasgow he takes his solitary walk in the corridors of the gaol; in the Millbank Penitentiary and at Lausanne, the prisoners meet for exercise in the open air, but silence is then strictly enforced. The prisoner is visited daily by the different officers of the gaol, who are expected to observe a considerate and kind manner towards those under their charge, and corporal punishment is unknown. By these means the inmates are brought to expect with pleasure the visits of their gaolers, and that spirit of hostility so often observed in those confined in other prisons towards their officers is here hardly ever to be met with. It appears obvious that, in prisons regulated under either of these two recent systems, the causes of the unequal rate of mortality must be sought for in the difference of the discipline in operation, and in the nature of the means by which that discipline is maintained.

The disorders most prevalent in prisons are obstructions of the intestinal canal, dropsy, phthisis pulmonalis, scrofula, diarrhœa, rheumatism, hysteria, &c. &c., and of all these, chronic thoracic disease is undoubtedly the most prevalent. According to Dr. Bâche of Cherry Hill Prison (United States), three fourths of the deaths in that establishment proceed from this cause alone, and of these 37 per cent. died of phthisis pulmonalis. It is worthy of remark that the mortality from this last-named disease is much more serious under the Auburn than under the Philadelphia system. Coindet suggests that the strict silence enjoined under the former plan may have a most deleterious influence upon the organs of respiration of those subjected to it, and also in a less direct manner upon their whole digestive system. But the perpetual seclusion from the open air, as is practised at Cherry Hill and Pittsburg (United States), cannot fail also to prove highly favorable to the development of thoracic disease. It is singular that the epidemic cholera never attacked the inmates of the prisons conducted on the Philadelphia systems, while it raged with violence in many of the others.

But the most important charge hitherto brought forward against the recent systems is that of increasing greatly the number of insane persons among the inmates of the gaols where they have been adopted. The complete silence enjoined under the Auburn system would of itself no doubt exercise a most debilitating influence upon the intellect; but we should remember that ample occupation for the mind is provided by a plentiful supply of appropriate books, and that a considerable portion of the day is spent in manual labour. Besides it is now pretty generally acknowledged that this perfect silence cannot be maintained, and the real origin of the numerous mental disorders in the Auburn prisons will perhaps be found in the severe discipline employed to enforce the observance of the silent plan.

With respect to the Philadelphia system, it has been urged that man is a social being formed to live in society, and that if he be long deprived of the benefit of this natural law he must necessarily suffer greatly both in his mental and corporal faculties. But the system of Philadelphia is not based on the principle of total seclusion, for all that is required is that, to avoid moral contamination, each prisoner shall be kept strictly separate from his fellow-captives. Here, as in the Auburn system, each prisoner is daily visited by the governor, the chaplain, the physician, and by the other officers of the gaol. In the prisons of Glasgow, Cherry Hill, &c., each inmate is daily visited at least twelve times by different individuals. "We must therefore be careful," says Professor Holst, "to draw the line of distinction between *separate* confinement and *solitary* confinement."

When the Philadelphia system was first adoped, it was justly objected to it that no manual labour was enjoined to the inmates of the prisons where the plan was pursued. This was unquestionably a serious oversight on the part of the projectors of the system; but the necessity for introducing manual labour soon became so apparent that it was universally enforced in the Philadelphia prisons, and in all others conducted under that system. Again it has been urged that separate confinement under this discipline must tend greatly to increase the habit of masturbation, which is now recognized as a frequent cause of mental disorder in young people. But it is evident from the prison reports that this crime is but too frequent in all places of confinement, nor can we discover that it prevails to a greater extent in the prisons of Philadelphia than in others under different discipline; while the evil consequence of prisoners occupying the same cell is well known to all who have investigated the records of the different gaols.

Professor Holst then proceeds to investigate at considerable length the reports furnished from the various prisons of Europe and America, in reference to the number of cases of insanity they present. Here his love for his favorite Philadelphia system has perhaps induced him to overstep the bounds of solid logical deduction. It is certain that in the American prisons cases of insanity are most numerous under the Philadelphia system; but Prof. Holst ventures to suggest that as but few or no retreats for the insane are provided in Pennsylvania, the more dangerous lunatics may be placed as a measure of safety within the walls of Cherry Hill or Pittsburg. The calculations of Coindet also, with respect to the prevalence of insanity in the penitentiary at Geneva, appear to our author to rest upon insecure and unsatisfactory data. Finally, in favour of the Philadelphia system Professor Holst produces the testimony of the commission appointed in 1837-8 by the senate of Pennsylvania to enquire into the influence of the recent systems upon the mental powers of the prisoners. It is here stated that after the most careful investigation, the prison of Cherry Hill presents as few if not fewer cases of insanity than any other prison of the United States, and that those instances of mental disorder which have occurred can be occasionally traced to other causes than the plan of discipline pursued.

During the twenty-five years that Mr. Brebner has been governor of the Glasgow Bridewell not a single case of insanity has occurred, although during that period of time from 40,000 to 50,000 prisoners have been confined within its walls, and in this gaol the Philadelphia system is enforced in the most complete possible form. Here Professor Holst seems to have forgotten that for at least twelve out of the twenty-five years above named the Philadelphia system was not in existence; but at all events it is hereby proved in the plainest manner that the introduction of that system has not increased the number of cases of insanity. The essay of Professor Holst concludes with a brief notice of the various prisons in which these several systems are pursued. Twenty-seven prisons in North America are regulated after the recent plans, seventeen being upon the Auburn system, which has been in operation since 1816, and ten upon that of Philadelphia, which was established in 1829. This latter discipline has also been adopted since 1834 in the Penitentiary of Lausanne in Switzerland, and at Warsaw in 1835 on the modified plan of Count Skarbeck. In France and Belgium the Philadelphia system has been as yet introduced only into a portion of the gaol at Ghent, and into the *model prison* at Paris. In this latter place it was first tried experimentally in that portion of the gaol allotted to children sent thither at the request of their parents (à la correction paternelle), and its good effects having soon appeared, it was adopted in all the other departments of that prison. In Great Britain and Ireland the Auburn system was adopted in 1835, but in 1839 the more recent system of Philadelphia was recommended by the parliamentary commissioners.

It is intended shortly to establish an experimental prison in Christiania in Norway for 100 prisoners, and which will be conducted on a plan very similar to that proposed by the general inspectors, Messrs. Crawford and Russell, and which is about to be tried on a large scale in the neighbourhood of London.

Professor Holst gives the decided preference to the Philadelphia system, but on a plan somewhat modified in its details as compared with the original.

"But," he continues, "while I declare my steady adherence to the Philadelphia system of prison discipline, I trust that I shall not be thought to uphold it in every particular, as it is enforced in the prison of Cherry Hill. I am not blind to the faults of this as well as of other systems. Within the last ten years the administration of prisons has made wonderful progress towards perfection, and the experience acquired and the researches entered into during that period have greatly aided to free the system from many of its faults, and to supply many of its deficiencies. This system (the Philadelphian), which had its origin in Europe, has gained immeasurably by its transit to America; and let us hope it may yet improve more fully when it returns to be readopted in the land of its birth. Two chief improvements I would venture to suggest, and in these I doubt not but that I shall be supported by the voice of the whole medical world: *first*, that the prisoners be allowed *daily* exercise in the open air; and *secondly*, that the period of their punishment be as much as is possible curtailed." (p. 30.)

---

